# Supplementary material for: Tensor decomposition of stimulated monocyte and macrophage gene expression profiles identifies neurodegenerative disease-specific trans-eQTLs
Source: PLoS Genet. 2020 Feb 3;16(2):e1008549. doi: 10.1371/journal.pgen.1008549 (PMC7018232; doi:10.1371/journal.pgen.1008549)
Supplement: S1 Fig — A: Shown in the heat map are −log10 transformed P-value of association between CG component individual scores and known covariates. Left: All 500 Components, Right: Sparse Components. B: For these sparse components, the component tissue scores were grouped based on activity in monocytes (MC) or macrophages (MP). (PDF) [file pgen.1008549.s001.pdf]

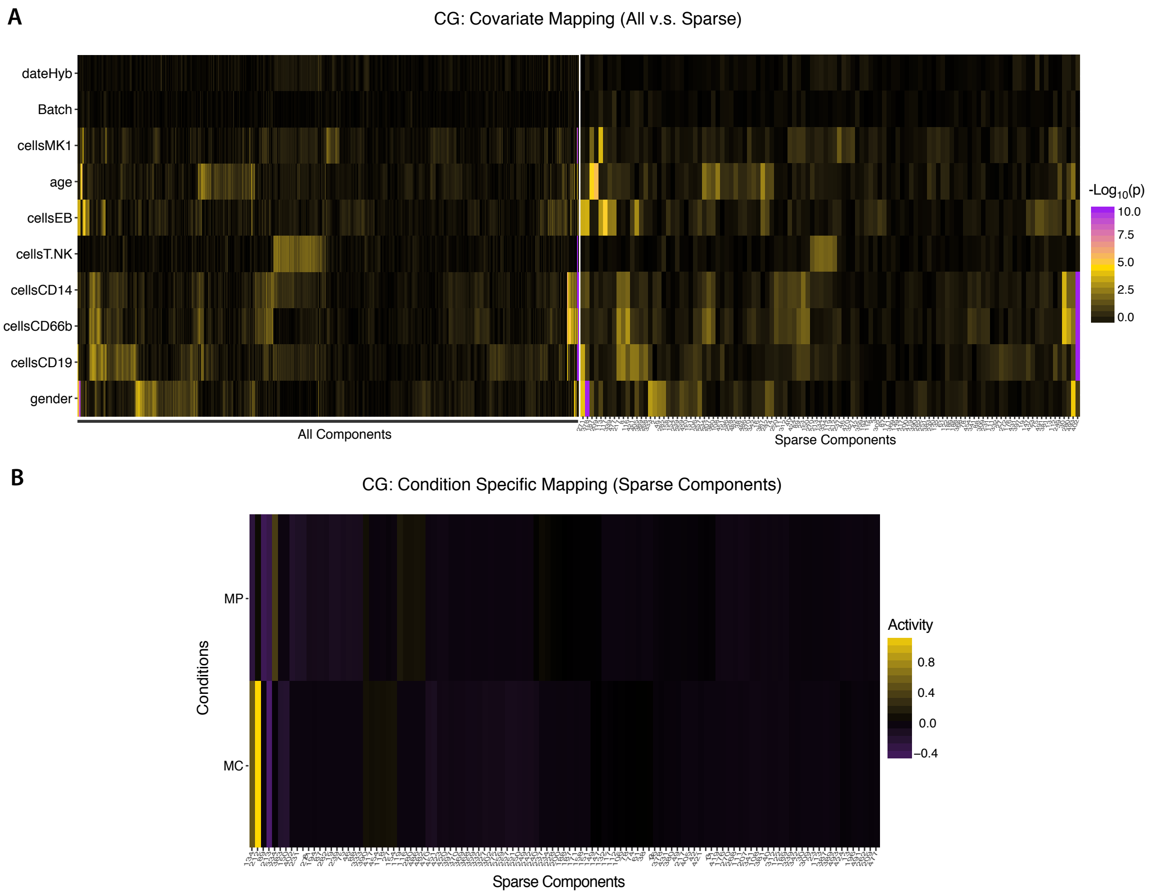

S1 Fig. Association of component individual scores with biological and technical covariates in Cardiogenics (CG). A: Shown in the heat map are $-log_{10}$ transformed P-value of association between CG component individual scores and known covariates. *Left:* All 500 Components, *Right:* Sparse Components. B: For these sparse components, the component tissue scores were grouped based on activity in monocytes (MC) or macrophages (MP).
