# Supplementary material for: Tensor decomposition of stimulated monocyte and macrophage gene expression profiles identifies neurodegenerative disease-specific trans-eQTLs
Source: PLoS Genet. 2020 Feb 3;16(2):e1008549. doi: 10.1371/journal.pgen.1008549 (PMC7018232; doi:10.1371/journal.pgen.1008549)
Supplement: S2 Fig — The SNP rs2275888 maps to FF component 337 enriched with type 1 interferon-related genes with a p-value = 1.7x10^−9^ and FDR = 1x10^−3^ and gene network similar to Fairfax et. al (2014) study. A cis-eQTL at rs2275888 for IFNB1 is associated with the expression of 17 genes in trans after 24-hour LPS stimulation, many of which are interferon response genes. (PDF) [file pgen.1008549.s002.pdf]

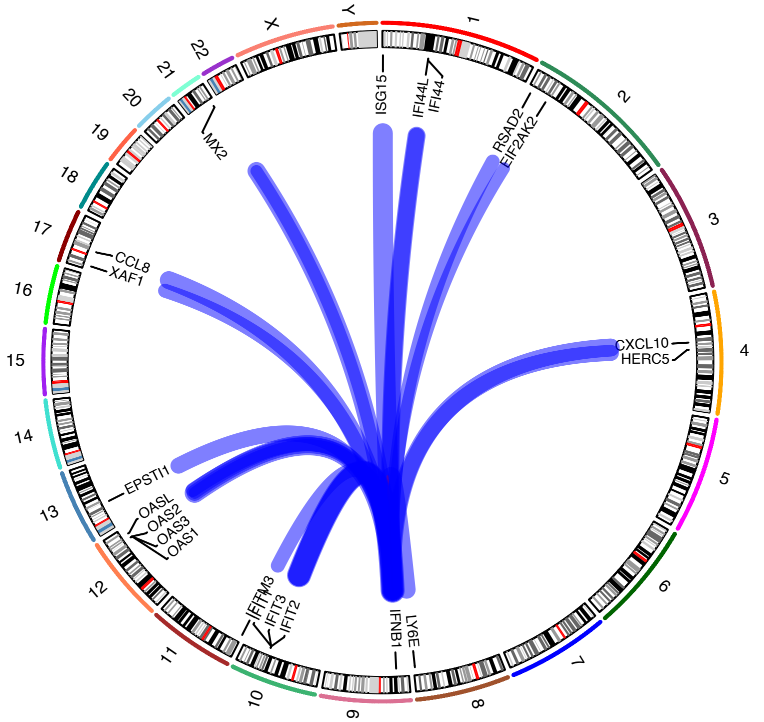

FF: Component 337

S2 Fig. Replication of previously identified *trans*-eQTLs in Fairfax *et. al* (2014). The SNP rs2275888 maps to FF component 337 enriched with type 1 interferon-related genes with a p-value $= 1.7 \times 10^{-9}$ and FDR $= 1 \times 10^{-3}$ and gene network similar to Fairfax et. al (2014) study. A *cis*-eQTL at rs2275888 for *IFNB1* is associated with the expression of 17 genes in *trans* after 24-hour LPS stimulation, many of which are interferon response genes.
