# Supplementary material for: Tensor decomposition of stimulated monocyte and macrophage gene expression profiles identifies neurodegenerative disease-specific trans-eQTLs
Source: PLoS Genet. 2020 Feb 3;16(2):e1008549. doi: 10.1371/journal.pgen.1008549 (PMC7018232; doi:10.1371/journal.pgen.1008549)
Supplement: S3 Fig — A: The gene expression profiles of component (363) with MHC and non-MHC genes in FF are shared among all stimuli (Top panel). Shown are the gene scores for the genes with Posterior Inclusion Probability (PIP) > 0.5 (bottom panel). Trans-eQTL for the MHC component co-localize with B) cholesterol associated risk variant rs9378212, C) Type 2 Diabetes risk variant rs9268645, and D) Coronary Artery Disease risk variant rs9268402. (PDF) [file pgen.1008549.s003.pdf]

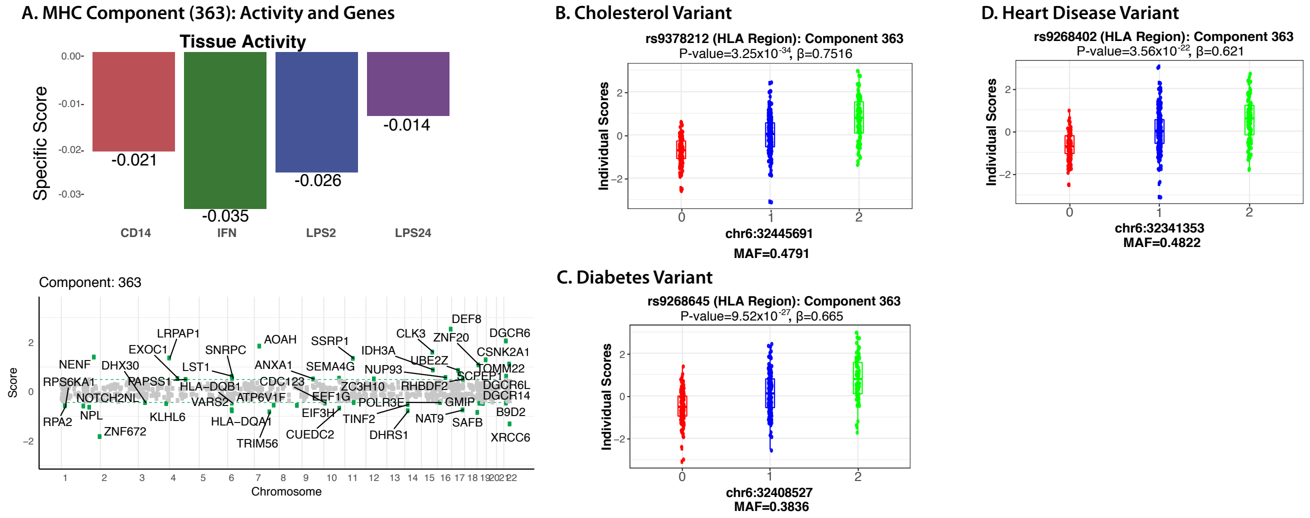

S3 Fig. *Trans*-eQTL mapping to a component with MHC and non-MHC genes. A: The gene expression profiles of component (363) with MHC and non-MHC genes in FF are shared among all stimuli (Top panel). Show are the gene scores for the genes with Posterior Inclusion Probability (PIP) $>$ 0.5 (bottom panel). *Trans-eQTL* for the MHC component co-localize with B) cholesterol associated risk variant $rs9378212$, C) Type 2 Diabetes risk variant $rs9268645$, and D) Coronary Artery Disease risk variant $rs9268402$.
