# Supplementary material for: Tensor decomposition of stimulated monocyte and macrophage gene expression profiles identifies neurodegenerative disease-specific trans-eQTLs
Source: PLoS Genet. 2020 Feb 3;16(2):e1008549. doi: 10.1371/journal.pgen.1008549 (PMC7018232; doi:10.1371/journal.pgen.1008549)
Supplement: S4 Fig — A: Cholesterol variant rs9378212 mediates the trans-effects through HLA-DQA1, B: Type 2 Diabetes variant rs9268645 mediates the trans-effects through HLADQA1, and C: Coronary Artery Disease variant rs9268402 mediates the trans-effects through HLA-DRB1. (PDF) [file pgen.1008549.s004.pdf]

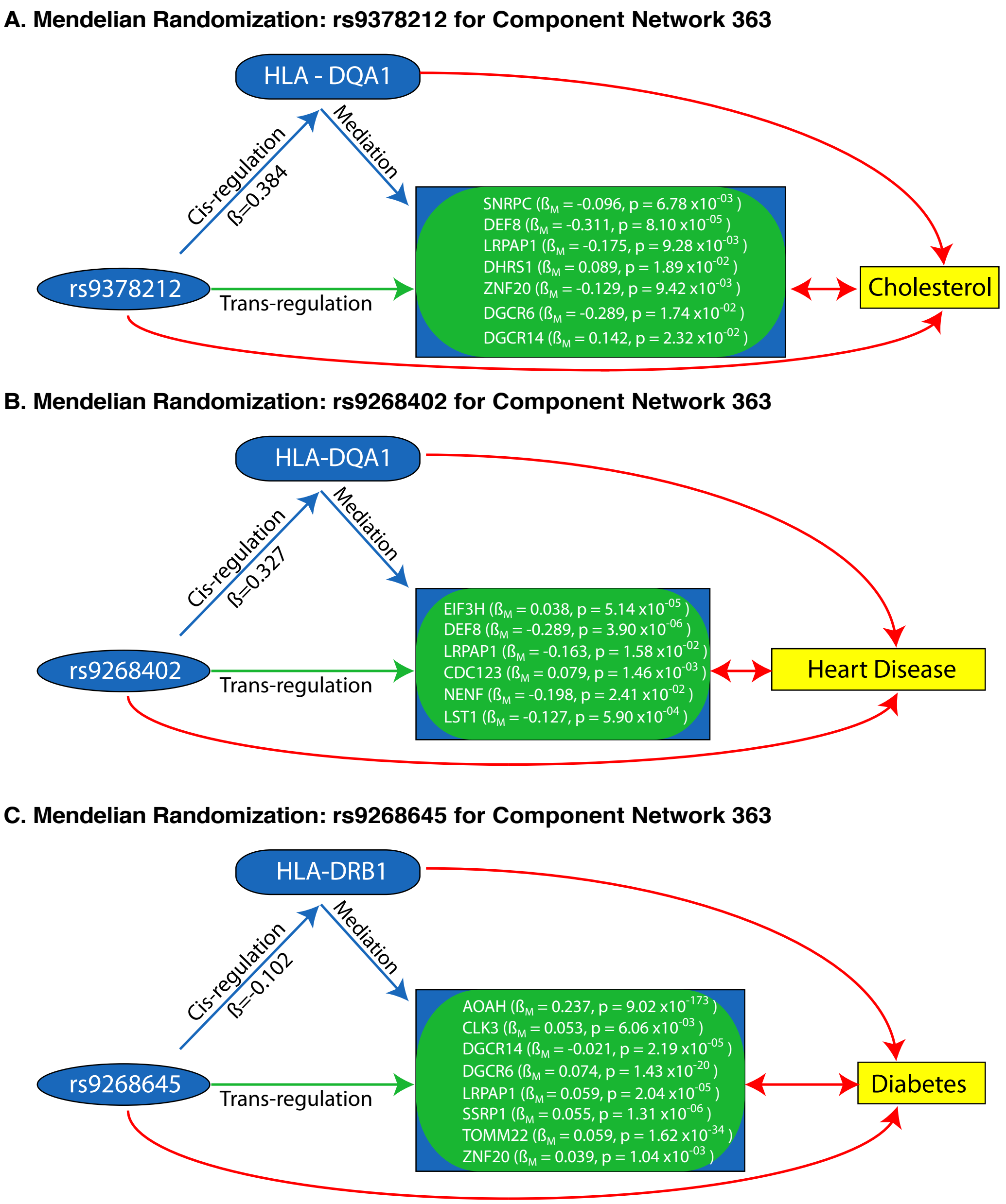

S4 Fig. Mendelian randomization analysis for *Trans*-eQTLs mediated by a *cis*-gene in the MHC. A: Cholesterol variant *rs*9378212 mediates the *trans*-effects through *HLA-DQA1*, B: Type 2 Diabetes variant *rs*9268645 mediates the effects through *HLA-DQA1*, and C: Coronary Artery Disease variant *rs*9268402 mediates the effects through HLA-DRB1.
