# Supplementary material for: Tensor decomposition of stimulated monocyte and macrophage gene expression profiles identifies neurodegenerative disease-specific trans-eQTLs
Source: PLoS Genet. 2020 Feb 3;16(2):e1008549. doi: 10.1371/journal.pgen.1008549 (PMC7018232; doi:10.1371/journal.pgen.1008549)
Supplement: S5 Fig — A: Top: Component 391 active in FFLPS24 (left) maps to Crohn’s disease associated variant rs503734 (Middle). The component contains genes from the members of the Zinc finger family (right). Bottom: The trans-eQTL is replicated in CG. B: (Top:) FF Component 105 active in FFLPS2 (left) maps to Coronary Artery Disease variant rs589448 (middle) and component gene scores (right). Bottom: The trans-eQTL is replicated in CG (Component 417 in CGMP). (PDF) [file pgen.1008549.s005.pdf]

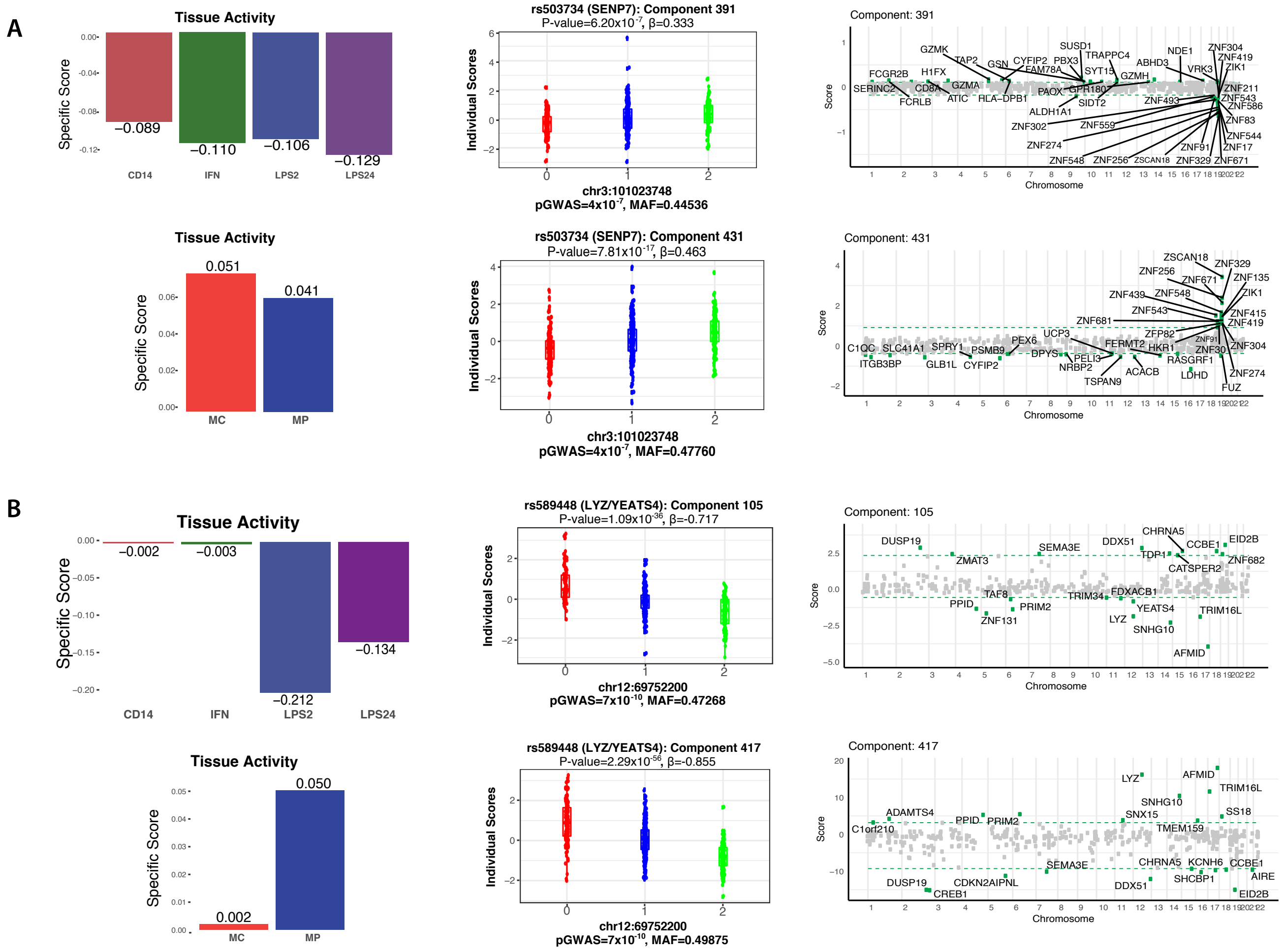

S5 Fig. Replication of *trans*-eQTLs that co-localize with disease-associated susceptibility allele. A: *Top:* Component 391 active in FF_{LPS24} (left) maps to Crohn's disease associated variant $rs503734$ (Middle). The component contain genes from the members of the Zinc finger family (right). *Bottom:* The *trans*-eQTL is replicated in CG. Component 431 in CG_{MP} (left) maps to the same Crohn's variant (middle). Zinc finger genes shows up in both datasets (right). B: (*Top:*) FF Component 105 active in FF_{LPS2} (left) maps to Coronary Artery Disease variant $rs589448$ (*middle*) and component gene scores (right). *Bottom:* The *trans*-eQTL is replicated in CG (Component 417 in CG_{MP} for the same variant $rs589448$ is a *cis*-eQTL to both *LYZ* and *YEATS4* in both FF and CG.
