# Supplementary material for: Tensor decomposition of stimulated monocyte and macrophage gene expression profiles identifies neurodegenerative disease-specific trans-eQTLs
Source: PLoS Genet. 2020 Feb 3;16(2):e1008549. doi: 10.1371/journal.pgen.1008549 (PMC7018232; doi:10.1371/journal.pgen.1008549)
Supplement: S6 Fig — (A) Crohn’s variant rs503734 mediate trans-effects through cis-gene SENP7 and (B) Coronary Artery Disease variant rs589448 mediate trans-effects through cis-gene LYZ and YEATS4. (PDF) [file pgen.1008549.s006.pdf]

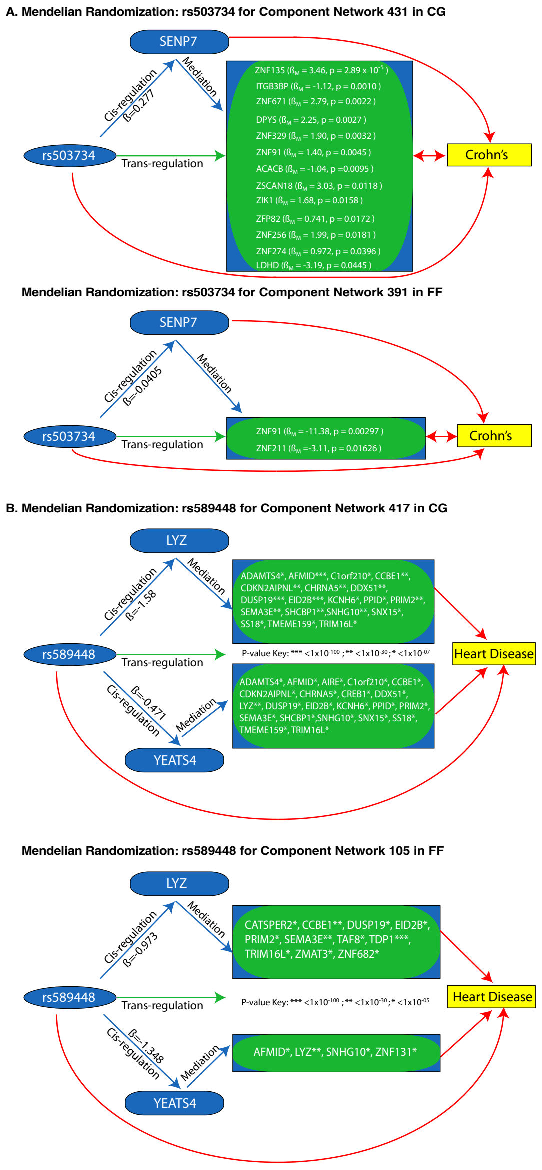

S6 Fig. Mendelian randomization analysis for disease-associated *trans*-eQTLs in FF and CG. (A) Crohn's variant *rs*503734 mediate *trans*-effects through cis-gene *SENP7* and (B) Coronary Artery Disease variant *rs*589448 mediate *trans*-effects through *cis*-gene *LYZ* and *YEATS4*.
