# Supplementary figures and images for: Tensor decomposition of stimulated monocyte and macrophage gene expression profiles identifies neurodegenerative disease-specific trans-eQTLs
Source: PLoS Genet. 2020 Feb 3;16(2):e1008549. doi: 10.1371/journal.pgen.1008549 (PMC7018232; doi:10.1371/journal.pgen.1008549)

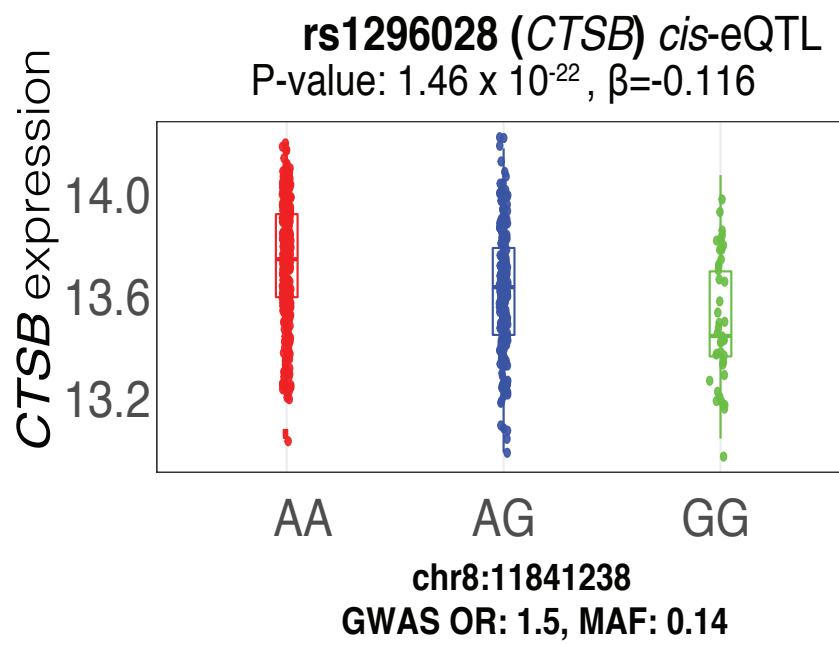

**S7 Fig.** *Cis*-eQTL (*rs1296028-CTSB*) co-localizes with Parkinson's disease associated variant *rs1296028*.

Supplement: S7 Fig — (PDF) [file pgen.1008549.s007.pdf]

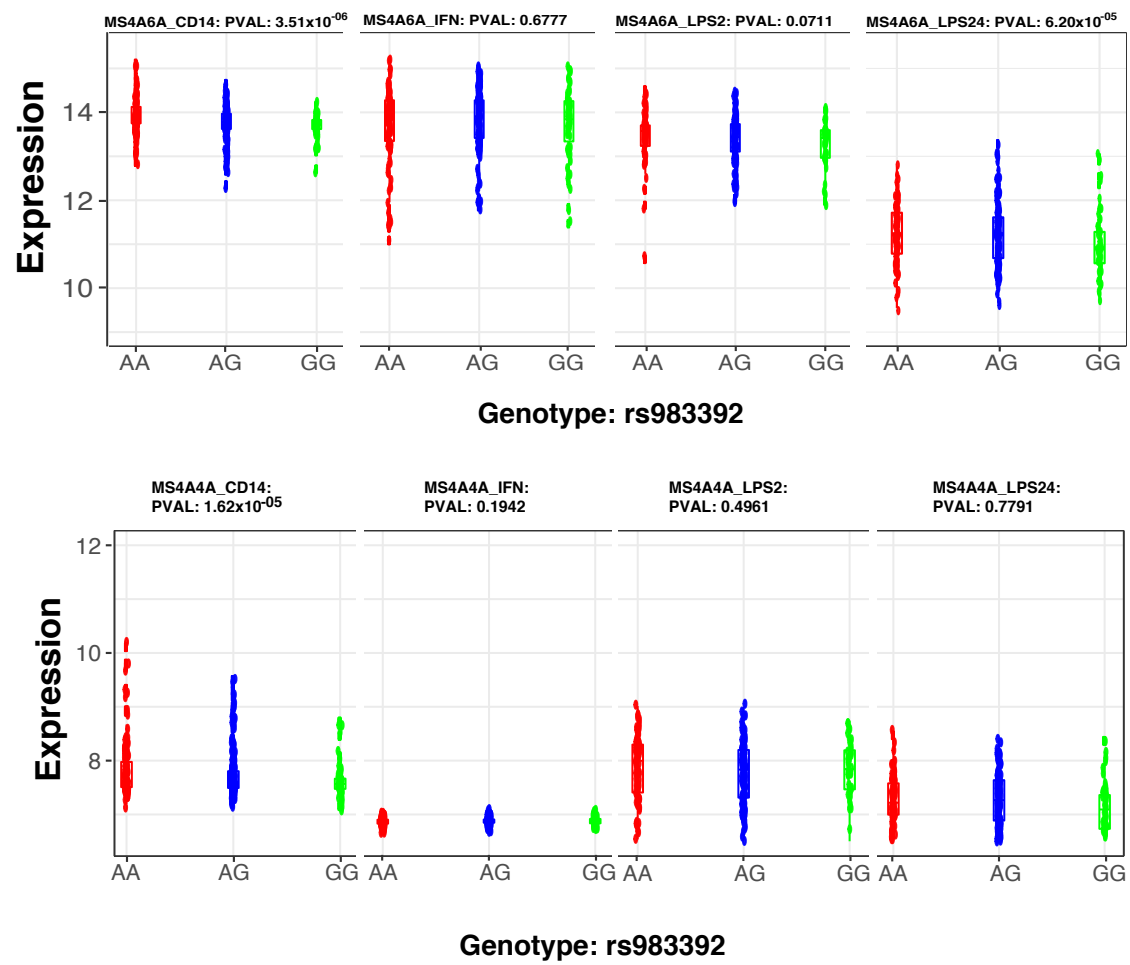

S11 Fig. Significant *cis*-eQTL effect for rs983392 to both *MS4A4A* and *MS4A6A* in baseline monocytes.

Supplement: S11 Fig — (PDF) [file pgen.1008549.s011.pdf]
