# Supplementary material for: Tensor decomposition of stimulated monocyte and macrophage gene expression profiles identifies neurodegenerative disease-specific trans-eQTLs
Source: PLoS Genet. 2020 Feb 3;16(2):e1008549. doi: 10.1371/journal.pgen.1008549 (PMC7018232; doi:10.1371/journal.pgen.1008549)
Supplement: S8 Fig — Shown are box plots for Parkinson’s disease associated variant rs1296028 (near CTSB) mapping to AARSD1, DDHD2, DGCR5, and DOK7. (PDF) [file pgen.1008549.s008.pdf]

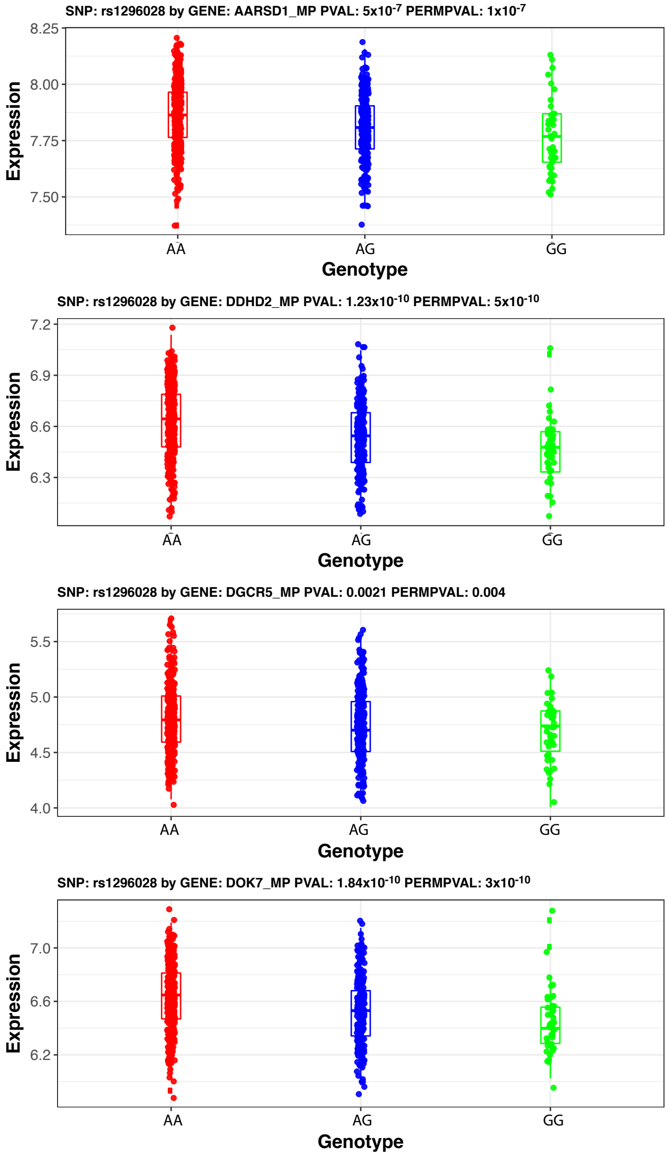

S8 Fig. SNP by Gene *trans*-eQTL association for *CG* Component 46. Shown are box plots for Parkinson's disease associated variant *rs*1296028 (near *CTSB*) mapping to *AARSD1, DDHD2, DGCR5, and DOK7*
