# Supplementary material for: Tensor decomposition of stimulated monocyte and macrophage gene expression profiles identifies neurodegenerative disease-specific trans-eQTLs
Source: PLoS Genet. 2020 Feb 3;16(2):e1008549. doi: 10.1371/journal.pgen.1008549 (PMC7018232; doi:10.1371/journal.pgen.1008549)
Supplement: S10 Fig — SNP by Gene association analysis was performed in an independent macrophage data from the STARNET cohort. Shown here are the trans-eQTL for rs1296028 and selected trans genes in the CTSB component at FDR < 0.20. (PDF) [file pgen.1008549.s010.pdf]

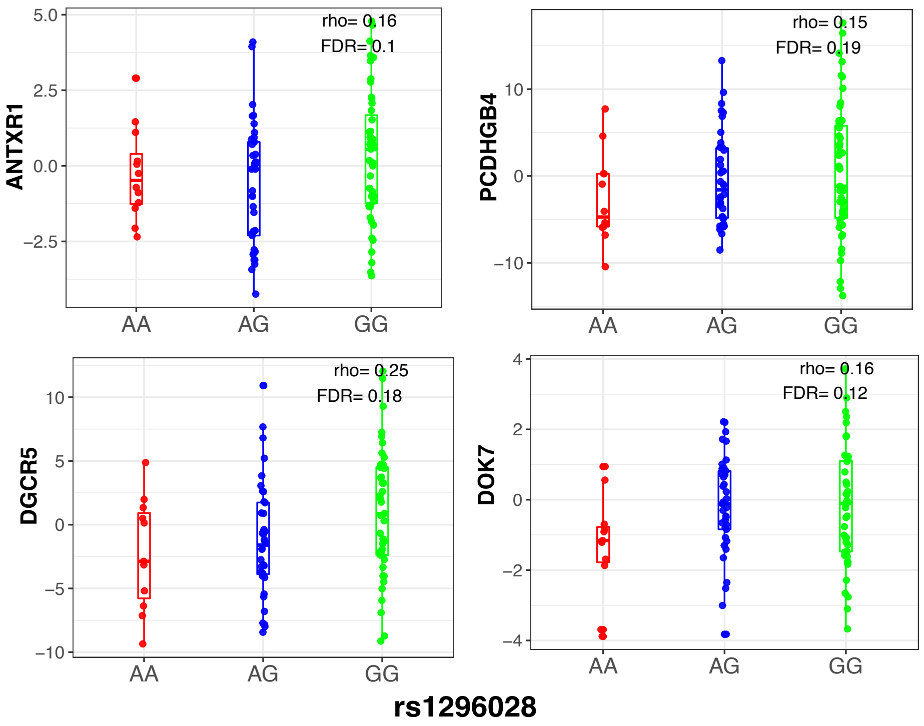

S10 Fig. Independent replication of *trans*-eGenes in the *CTSB* component. SNP by Gene association analysis was performed in an independent macrophage data from the STARNET cohort. Shown here are the trans-eQTL for rs1296028 and selected *trans* genes in the *CTSB* component at $FDR < 0.20$.
