# Supplementary material for: Tensor decomposition of stimulated monocyte and macrophage gene expression profiles identifies neurodegenerative disease-specific trans-eQTLs
Source: PLoS Genet. 2020 Feb 3;16(2):e1008549. doi: 10.1371/journal.pgen.1008549 (PMC7018232; doi:10.1371/journal.pgen.1008549)
Supplement: S12 Fig — FF Component 26 trans-eGenes: CD38, CUL1, CYP4F3, FAM129B, and FBXO6; trans-eSNP rs983392. (PDF) [file pgen.1008549.s012.pdf]

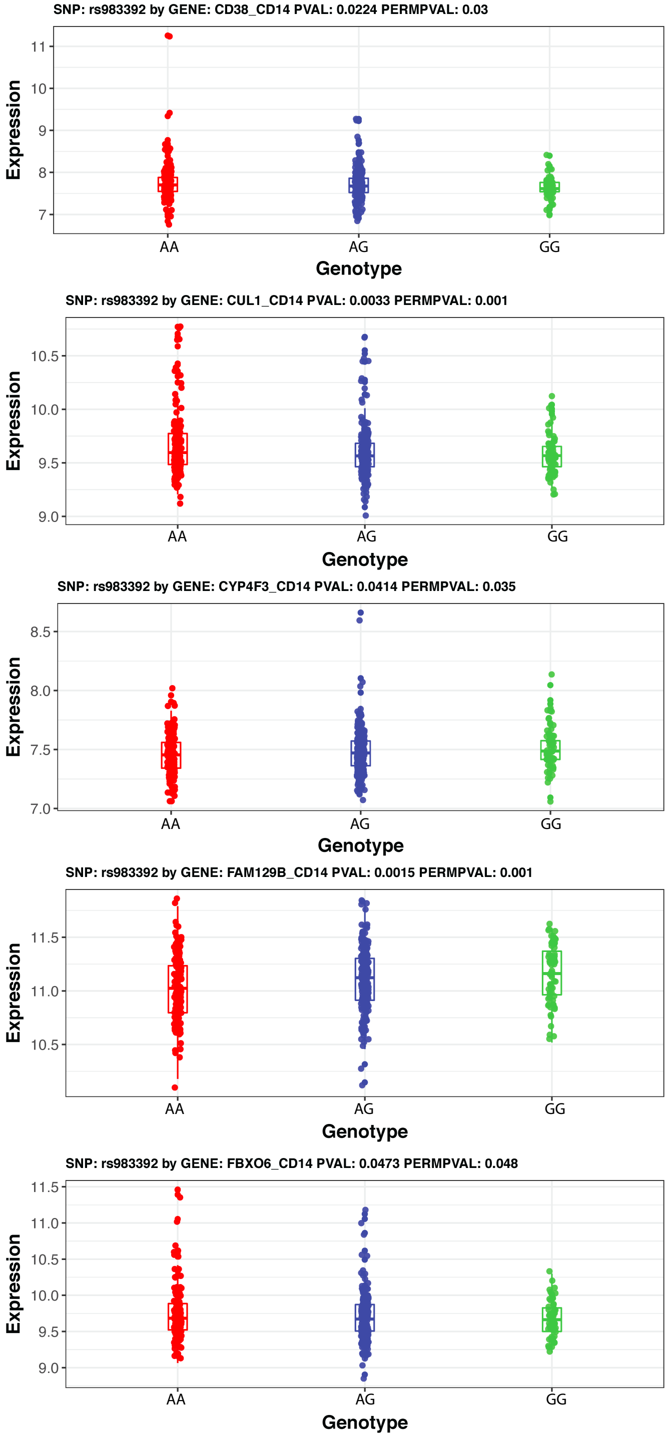

S12 Fig. FF **Component** 26 **trans-eGenes:** ***CD38, CUL1, CYP4F3, FAM129B, and FBXO6*****; SNP by Gene in** FF_{CD14} **for Alzheimer's variant** $rs983392$**.**
