# Supplementary material for: Tensor decomposition of stimulated monocyte and macrophage gene expression profiles identifies neurodegenerative disease-specific trans-eQTLs
Source: PLoS Genet. 2020 Feb 3;16(2):e1008549. doi: 10.1371/journal.pgen.1008549 (PMC7018232; doi:10.1371/journal.pgen.1008549)
Supplement: S13 Fig — FF Component 26 trans-eGenes: GBP1, HMGCR, LGALS3BP, PADI4 and PSME2; trans-eSNP rs983392. (PDF) [file pgen.1008549.s013.pdf]

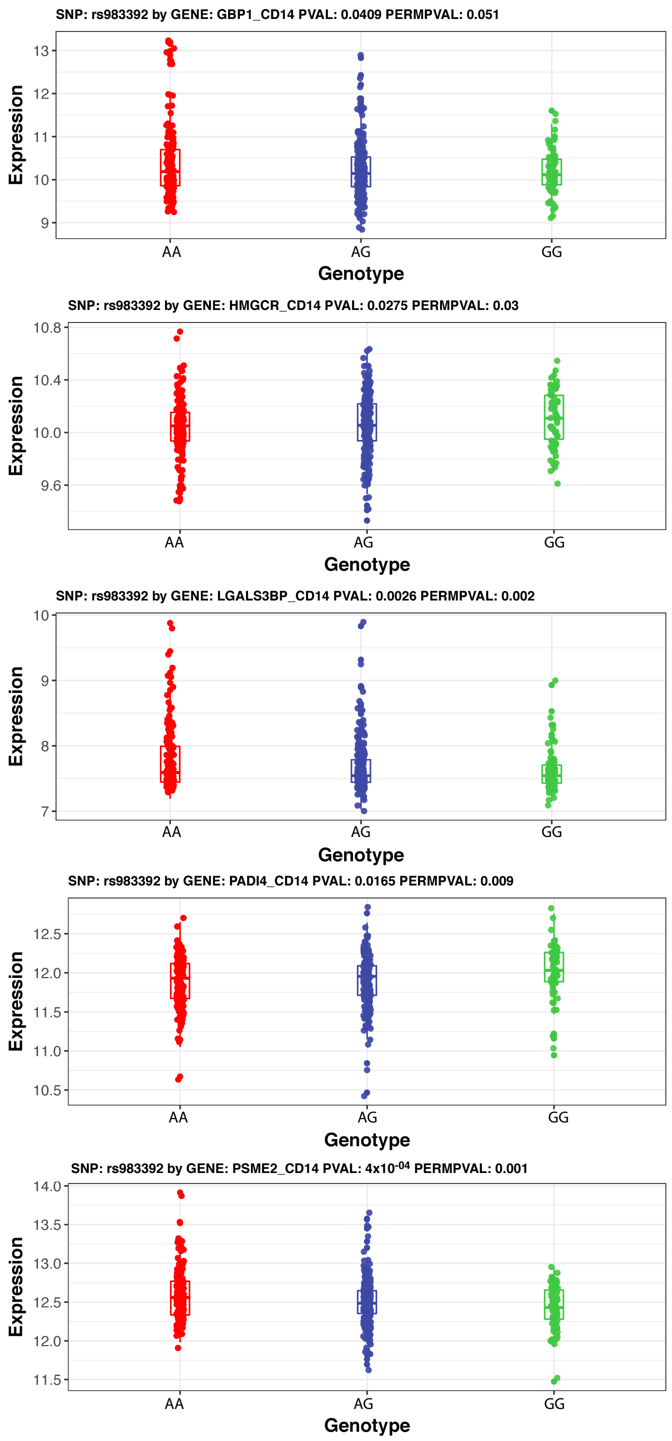

S13 Fig. FF **Component 26 trans-eGenes:** ***GBP1, HMGCR, LGALS3BP, PADI4 and PSME2*****; SNP by Gene in** FF_{CD14} **for Alzheimer's variant** $rs983392$
