# Supplementary material for: Tensor decomposition of stimulated monocyte and macrophage gene expression profiles identifies neurodegenerative disease-specific trans-eQTLs
Source: PLoS Genet. 2020 Feb 3;16(2):e1008549. doi: 10.1371/journal.pgen.1008549 (PMC7018232; doi:10.1371/journal.pgen.1008549)
Supplement: S14 Fig — FF Component 26 trans-eGenes: QPCT, SERPING1, STAT1, STOM, SYTL3, and TAP1; trans-eSNP rs983392. (PDF) [file pgen.1008549.s014.pdf]

SNP: rs983392 by GENE: QPCT_CD14 PVAL: 0.0016 PERMPVAL: 0.002

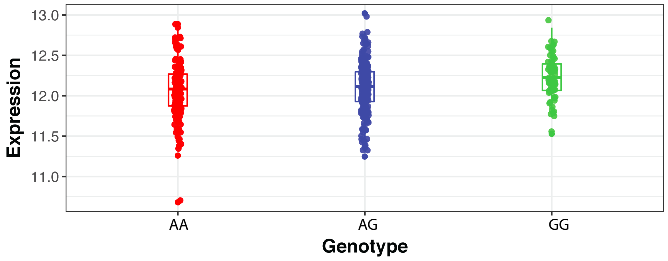

SNP: rs983392 by GENE: SERPING1_CD14 PVAL: 0.0454 PERMPVAL: 0.037

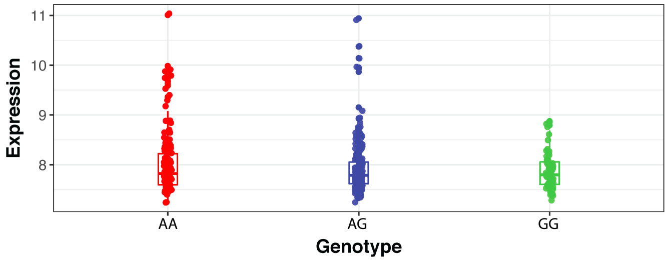

SNP: rs983392 by GENE: STAT1_CD14 PVAL: 0.0427 PERMPVAL: 0.047

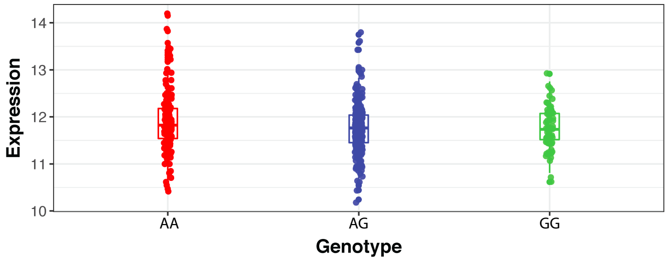

SNP: rs983392 by GENE: STOM_CD14 PVAL: 0.006 PERMPVAL: 0.009

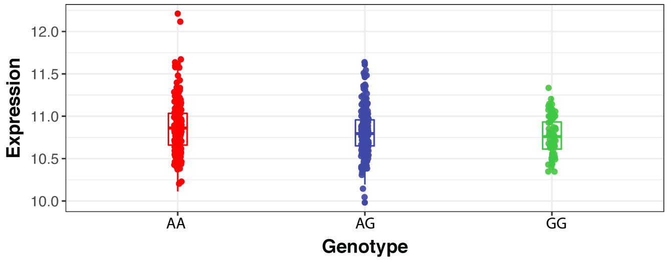

SNP: rs983392 by GENE: SYTL3_CD14 PVAL: 0.0158 PERMPVAL: 0.013

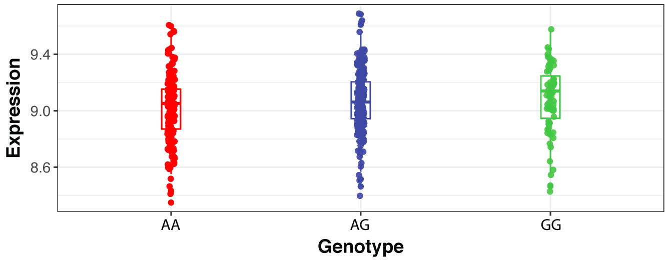

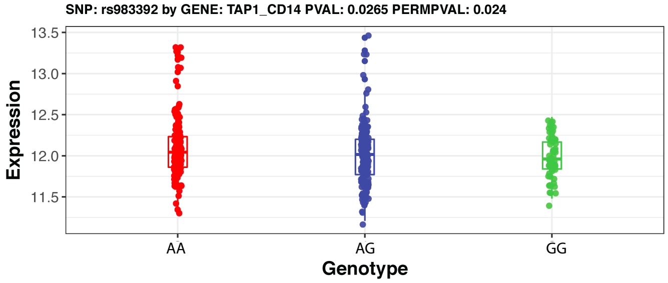

S14 Fig. FF **Component** 26 **trans-eGenes:** ***QPCT, SERPING1, STAT1, STOM, SYTL3, and TAP1***; **SNP by Gene in** FF_{CD14} **for Alzheimer's variant** $rs983392$
