# Supplementary material for: Tensor decomposition of stimulated monocyte and macrophage gene expression profiles identifies neurodegenerative disease-specific trans-eQTLs
Source: PLoS Genet. 2020 Feb 3;16(2):e1008549. doi: 10.1371/journal.pgen.1008549 (PMC7018232; doi:10.1371/journal.pgen.1008549)
Supplement: S16 Fig — FF Component 22 trans-eGenes: ISG20, ITGAX, MT1E, MT1F, and OASL; trans-eSNP rs9331896. (PDF) [file pgen.1008549.s016.pdf]

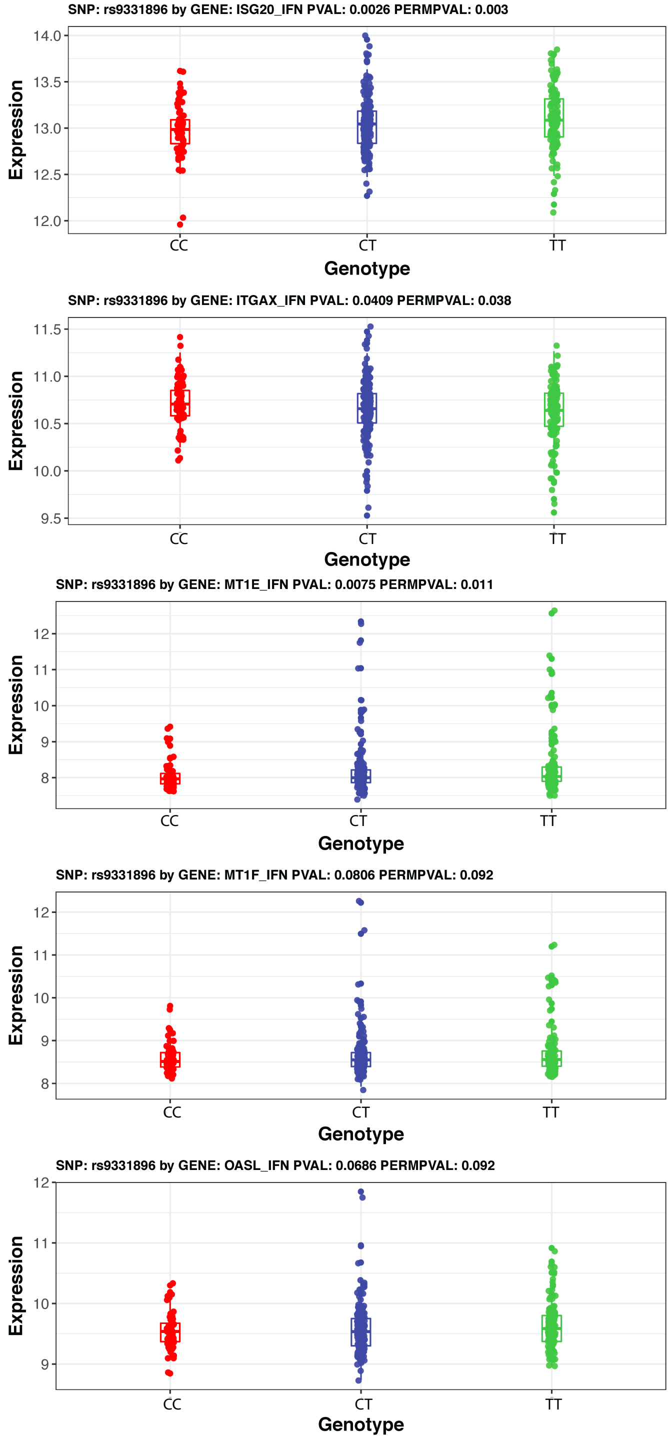

S16 Fig. FF **Component** 22 **trans-eGenes:** ***ISG20, ITGAX, MT1E, MT1F, and OASL*****; SNP by Gene in** FF_{IFN} **for Alzheimer's variant** $rs9331896$
