# Supplementary material for: Tensor decomposition of stimulated monocyte and macrophage gene expression profiles identifies neurodegenerative disease-specific trans-eQTLs
Source: PLoS Genet. 2020 Feb 3;16(2):e1008549. doi: 10.1371/journal.pgen.1008549 (PMC7018232; doi:10.1371/journal.pgen.1008549)
Supplement: S17 Fig — FF Component 22 trans-eGenes: RSAD2, SLC2A5, TOP2B, and VASH1; trans-eSNP rs9331896. (PDF) [file pgen.1008549.s017.pdf]

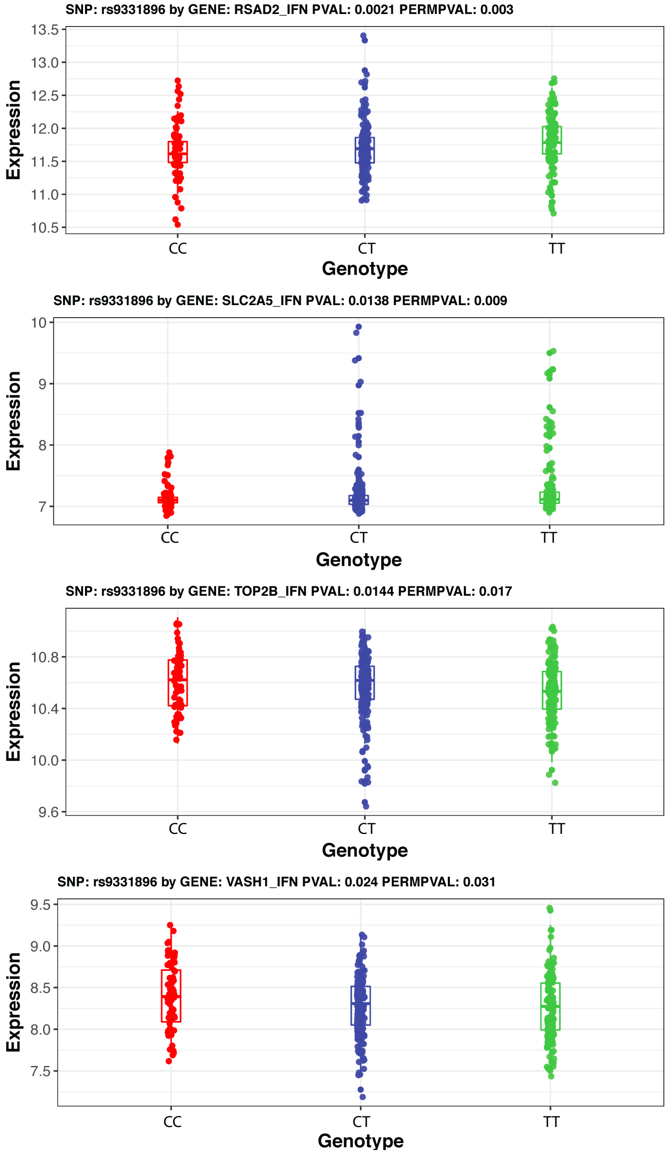

S17 Fig. FF **Component** 22 **trans-eGenes:** ***RSAD2, SLC2A5, TOP2B, and VASH1***; **SNP by Gene in** FF_{IFN} **for Alzheimer's variant** $rs9331896$
