# Supplementary material for: Tensor decomposition of stimulated monocyte and macrophage gene expression profiles identifies neurodegenerative disease-specific trans-eQTLs
Source: PLoS Genet. 2020 Feb 3;16(2):e1008549. doi: 10.1371/journal.pgen.1008549 (PMC7018232; doi:10.1371/journal.pgen.1008549)
Supplement: S18 Fig — The PPI suggests that the products of CLU physically interact with complement proteins. The PPI was generated with GeNets Meta network v1.0 database. (PDF) [file pgen.1008549.s018.pdf]

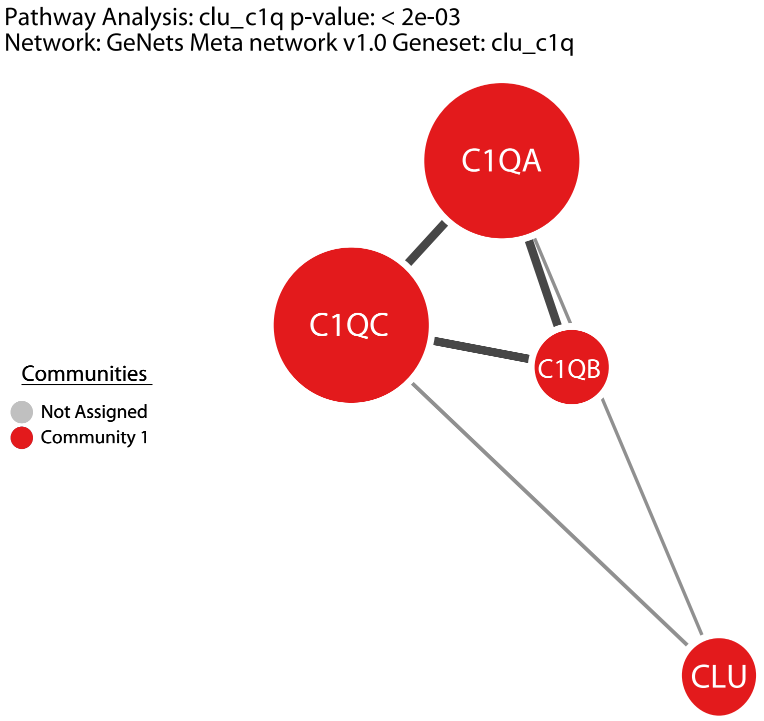

S18 Fig. Protein-protein interaction (PPI) network of protein products of *CLU, C1QA, C1QB and C1QC*. The PPI suggests that the products of CLU physically interacts with complement proteins.The PPI was generated with GeNets Meta network v1.0 database.
