# Supplementary material for: Tensor decomposition of stimulated monocyte and macrophage gene expression profiles identifies neurodegenerative disease-specific trans-eQTLs
Source: PLoS Genet. 2020 Feb 3;16(2):e1008549. doi: 10.1371/journal.pgen.1008549 (PMC7018232; doi:10.1371/journal.pgen.1008549)
Supplement: S19 Fig — SNP by Gene association analysis was performed in an independent baseline monocytes from the ImmVar cohort. Shown here are the trans-eQTL for rs983392 and selected trans genes in the MS4A4A/6A component (FDR < 0.20). (PDF) [file pgen.1008549.s019.pdf]

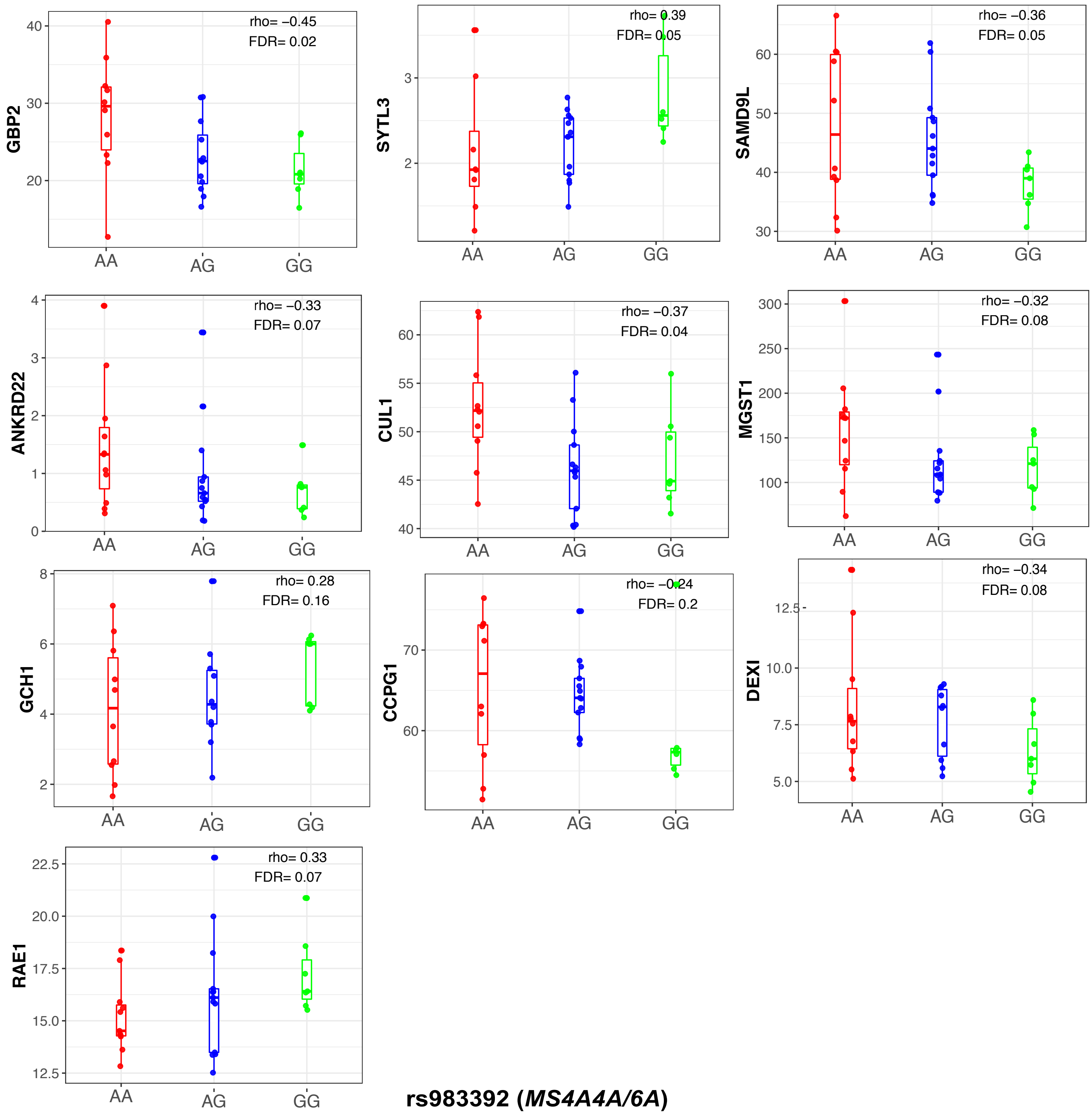

S19 Fig. Independent replication of *trans*-eGenes in the *MS4A4A/6A* component. SNP by Gene association analysis was performed in an independent baseline mono-

cytes from the ImmVar cohort. Shown here are the *trans*-eQTL for rs983392 and selected *trans* genes in the *MS4A4A/6A* component ($FDR < 0.20$).
