# Supplementary material for: Tensor decomposition of stimulated monocyte and macrophage gene expression profiles identifies neurodegenerative disease-specific trans-eQTLs
Source: PLoS Genet. 2020 Feb 3;16(2):e1008549. doi: 10.1371/journal.pgen.1008549 (PMC7018232; doi:10.1371/journal.pgen.1008549)
Supplement: S20 Fig — SNP by Gene association analysis was performed in an independent stimulated monocyte data from the ImmVar cohort. Shown here are the trans-eQTL for rs9331896 and selected trans genes in the CLU component (FDR < 0.20). (PDF) [file pgen.1008549.s020.pdf]

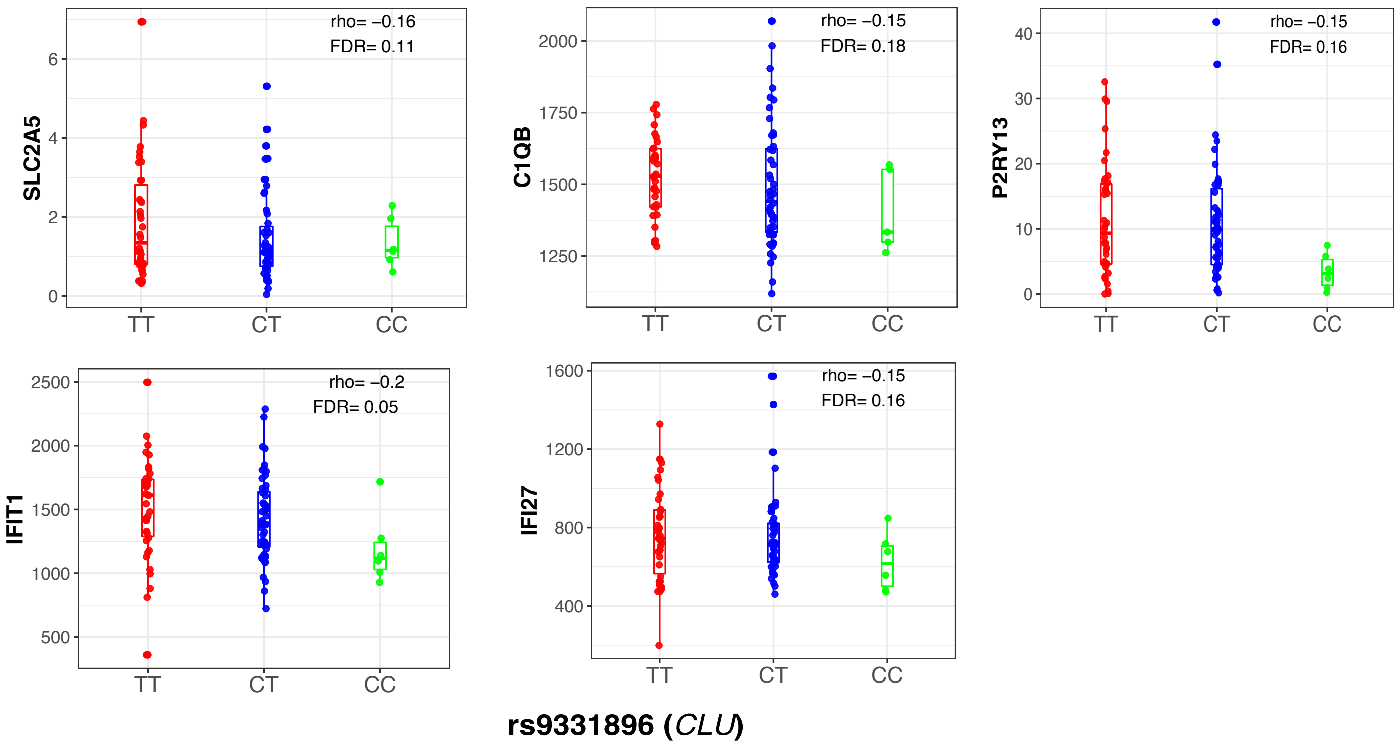

S20 Fig. Independent replication of *trans*-eGenes in the *CLU* component. SNP by Gene association analysis was performed in an independent stimulated monocytes from the ImmVar cohort. Shown here are the *trans*-eQTL for rs9331896 and selected *trans* genes in the *CLU* component ($FDR < 0.20$).
