# Supplementary material for: Tensor decomposition of stimulated monocyte and macrophage gene expression profiles identifies neurodegenerative disease-specific trans-eQTLs
Source: PLoS Genet. 2020 Feb 3;16(2):e1008549. doi: 10.1371/journal.pgen.1008549 (PMC7018232; doi:10.1371/journal.pgen.1008549)
Supplement: S1 Note — (PDF) [file pgen.1008549.s031.pdf]

Supplemental Notes 1

1. Assumptions and Sparsity Ranking Statistic Formulation.

Implementation assumptions: In a slight contrast to Hore *et al.,* we impose several strict assumptions prior to implementation. Assumption 1: The unobservable latent space captured by this model is independent for each run. We believe that each model run attempting to capture the latent or underlying space driving the multi-way expression data is independent of each other. Assumption 2: since sparsity is attempted by a spike slab prior driving some values close to (but not necessarily) zero, we use distribution cutoffs to determine thresholding. Since the genes are driven close to but not exactly to zero, top scoring distributional genes are major contributing factor to the network. We use 2.5% tail distributions. Assumption 3: sparsity as per assumption 2 has a maximum limit of T. The idea is that even with distributional thresholding per each component, noisy components may still have a large number of genes. This criterion will reduce their effect of admittance into further analysis. We define T to be 40.

Ranking Statistic Formulation: While sparsity is built into the model through the spike-slab prior, low scoring genes in a component are not truly zero but driven close to it. This makes it difficult to determine which component are truly sparse. In an attempt to measure sparsity, a ranking statistic was created. So, for $i = 1, ..., n$, n are number of components, let $R_i(w_i, N_i) = R_i = 1_{\{N_i \leq T\}} \times \frac{f(w_i)}{g(N_i)}$ where w_i is a weight function for the significant genes per component based on assumption 3, the functions f and g are mapping functions chosen to reduce the effect of high scoring component gene scores, N_i is the (non-zero) number of significant genes per component and the indicator function drives the ranking of components violating assumption 4 straight to zero. We have defined: $w_i = \frac{max_i|x_i| \exp(-(max_i|x_i|)^{-1})}{MAD_i(x_i)}$ where x_i are the actual significant gene scores. This weight function says weight by the largest score proportional to the median absolute deviation (MAD) (assumed to be non-zero) while simultaneously driving small maximum (magnitude) scores close to zero: $f(w_i) = w_i$ and

$g(N_i) = log(N_i)$. The next issue becomes how to choose a cut-off for this ranking statistic (apart from those driven directly to zero). To do this we propose the following cutoff with a bounding proof.

Proposed cut-off formula and value: Without attaching distributional assumptions to these functions, we instead propose a formula for cutoff per component then summarize it (through a limiting case). For a (non-zero) median absolute deviation, we let c be the (lower) bound cut-off, then for a given $y = max_i|x_i|$ or maximum absolute score value for that component with a threshold of N (N_i from before) or T (from assumption 3), $c(y, N) \geq \frac{1}{1.4826\exp(y^{-1})log(N)}$. So for the limiting case where $N = T = 40$, $\lim_{y\to\infty} = 0.1828443$ and so our cut-off value is 0.18.
